# The Role of Physical Education Lessons and Recesses in School Lifestyle of Adolescents

**DOI:** 10.1111/josh.12362

**Published:** 2016-01-13

**Authors:** Karel Frömel, Zbyněk Svozil, František Chmelík, Lukáš Jakubec, Dorota Groffik

**Affiliations:** a Faculty of Physical Culture, Palacký University Olomouctřída Míru 117, 77111 Olomouc, Czech Republic; b Faculty of Physical Culture, Palacký University Olomouctřída Míru 117, 77111 Olomouc, Czech Republic; c Faculty of Physical Culture, Palacký University Olomouctřída Míru 117, 77111 Olomouc, Czech Republic; d Faculty of Physical Culture, Palacký University Olomouctřída Míru 117, 77111 Olomouc, Czech Republic; e Department of Physical Education, The Jerzy Kukuczka Academy of Physical EducationUl. Mikolowska 72A, 40065 Katowice, Poland

**Keywords:** school physical activity, physical education, recess, health education, youth lifestyle

## Abstract

**BACKGROUND:**

This study investigates school lifestyle among adolescents in terms of physical activity (PA) structure: (1) adolescents participating in a physical education lesson (PEL) versus (2) aggregate recess time exceeding 60 minutes.

**METHODS:**

The research was conducted in 24 secondary schools in the Czech Republic (boys N = 208, girls N = 433). For the whole day (1-3 days) participants wore the ActiTrainer accelerometer, which monitored PA, and heart rate. A total of 1122 school days were recorded.

**RESULTS:**

Both boys and girls participating in a PEL reported significantly better results compared with nonparticipating individuals regarding all indicators of volume and intensity of school PA (SPA). In most SPA indicators, longer aggregate recess time (>60 minutes) had a statistically significant effect, particularly on the volume of SPA. The recommended 500 steps/hours for SPA was achieved by 83% of boys participating in PEL and 69% of girls. In contrast just 32% of nonparticipating boys and 31% of girls reached this level. With longer recess time the recommendation was met by 43% of boys (42% of girls) compared with 26% of boys (23% of girls) with shorter recess time.

**CONCLUSIONS:**

An increase in SPA and an improved lifestyle in adolescents on school days are significantly supported more by PELs than by longer recess time.

Education in secondary schools should meet the most rigorous requirements for a healthy lifestyle in adolescents because the process of education in school needs to be understood as a form of preparation for future occupation. Through a quality educational program, schools have an opportunity to ensure sufficient physical literacy in adolescents^1^ and to support their health-related fitness.^2^ School physical activity (SPA) is a significant component of daily PA.^3^,^4^ Higher school-day moderate-to-vigorous physical activity (MVPA) is associated with higher overall daily MVPA^5^,^6^ and has a significantly positive influence on children's mental health.^7^ More physically active children on 5 school days are also more physically active during classtime and lunchtime.^8^

The decisive role in SPA is played by physical education lessons (PELs) and recess time; these can make important contributions to children's overall PA,^9^–^12^ especially in combination with other school-based PA opportunities.^13^ There is sufficient evidence that on school days with PELs, both boys and girls have higher daily PA.^14^–^17^ Also, interventions can increase the proportion of time that students spend in MVPA during PELs.^18^ PELs should cover at least 50% of MVPA,^4^,^19^ fitness-oriented PA, and at least 2 considerable physiological impulses close to maximum or submaximum heart rate (HR).^20^

We have sufficient knowledge about recess time and lunchtime and their contribution to increased PA, particularly in children,^12^,^21^–^23^ and to a lesser extent, in secondary school adolescents.^24^,^25^ The recorded decrease in the proportion of PA during recess time and lunchtime in all-day PA after transition from primary to secondary schools^26^ emphasizes the significance of research and interventions in these schools. There is also insufficient knowledge about the type of PA and PA intensity during recess time.^11^,^27^ In addition, there is little knowledge about the association between the type of lesson and subsequent recess in terms of compensation for mental stress in adolescents.

There is nearly universal agreement on the recommendation of 60 minutes of PA per day for adolescents, mainly involving MVPA.^28^–^31^ Similarly, there is an agreement on the recommendation concerning the amount of daily PA in adolescents on school days of approximately 11,000-14,000 steps/day.^32^,^33^ Fewer recommendations are available for SPA. The most promoted and school-acknowledged recommendation is to ensure 30 minutes of PA, if PELs are not included in the daily school program.^4^ There is no agreement on recommendations for vigorous PA that schools are likely to promote in nonorganized activities for adolescents.

The aim of this study was to investigate the structure of PA in a school program: (1) adolescents participating in a PEL versus (2) aggregate recess time exceeding 60 minutes. The aim of the study is to answer the following research question: *During SPA*, *to what extent can PELs substitute for longer aggregate recess time*? The final aim is to draft a recommendation for minimum PA supporting a healthy lifestyle in adolescents.

## METHODS

### School Setting and Participants

The research was performed in the Czech Republic between 2012 and 2013 in 24 secondary schools in the locations of residence of PE teachers. PELS took 45 minutes at all participating schools. Schools followed the national framework program for PE for which they are allowed to enrich the program according to their own educational plans, the needs and interests of their students, and regional or local practices. The program also incorporates standards similar to what one would find in a K-12 curriculum in the United States and many other countries.

A recess of 10 minutes took place between lessons, with the exception of the 15-to-20-minute recess after the second lesson. In addition, if there was a recess for lunch, the recess was at least 30 minutes in duration to be in compliance with school policy requirements. During school recesses and lunch recesses, the participants could move freely on campus and have opportunity to use school facilities for various unstructured physical activities. None of the schools had implemented a special PA program intended for recesses, lunch recesses, or for carrying out lessons in subjects other than PE. However, all schools had extracurricular programs that involved PA.

The research involved a total of 208 boys and 433 girls (Table1). Overall, 91% of parents gave consent (student assent) to the research. Owing to an incomplete all-day HR record, 202 participants, and 2 participants with multiple-hour swimming training were excluded from the results. Boys and girls were divided into 2 groups: (1) according to participation in PEL; and (2) according to aggregate recess time ≤60 and >60 minutes. Participation of students in PELs was not modified. In all schools, PELs were delivered in a differentiated way, ie, specific activities for boys versus girls. The aggregate recess time included lunchtime. Only 6.15% of boys and 9.71% of girls were in the overweight or obese zone (≥85th percentile).

**Table 1:** Sample Characteristics

		Age (Years)		Weight (kg)		Height (cm)		BMI (kg/m^2^)		HR_rest_/min	
Characteristics	N	M	SD	M	SD	M	SD	M	SD	M	SD
Boys	208	16.55	1.27	70.48	12.69	178.15	8.36	22.20	3.76	60.63	7.29
Girls	433	16.59	1.15	59.26	9.58	166.98	6.41	21.22	2.95	63.64	7.00

BMI, body mass index; M, mean; HR_rest_, resting heart rate.

## Instrumentation

The monitoring device, ActiTrainer™ (Pensacola, FL) (http://www.theactigraph.com/products/actitrainer) is a combination of ActiGraph accelerometer and HR sensor Polar type. Our department verified the validity of ActiTrainer-based step counting in field conditions. The correlational values expressing the relationships between actual and device-measured steps ranged from .96 to .97.^34^ The monitoring days were selected according to computer room availability to ensure the participants' registration in the Internet-based Indares program (http://www.indares.com). Following a joint training session, the participants used the ActiTrainer accelerometer to monitor PA from the morning of the following day (after morning hygiene) throughout the whole day (except showering and swimming) until evening hygiene before going to bed plus 2 consecutive school days under the same measurement conditions. During the training session the participants were instructed how to wear the devices, how to measure HR, how to use the Internet-based Indares program, and how to use additional record sheets (course of the day, recording PA duration, and type). Data on age, height, and weight were taken from official school documents. Resting HR was measured by the participants individually according to precise instructions in the morning after wake-up repeatedly 3 times in 15-second intervals; HR-minute was recorded together with a 1-minute average value. The individually measured HR values were used to calculate an average value, which was, in the case of higher values, corrected according to the lowest daily HR value measured by the ActiTrainer device. The accuracy of HR monitoring was checked upon arrival in school using the Heart Rate Monitor Polar S610™ (Polar Electro Oy, Kempele, Finland). Apart from the ActiTrainer, the participants also wore the Digi-Walker SW-700 pedometer (Yamasa Tokei Keiki Co., LTD., Tokyo, Japan) for motivation and information reasons. To limit the effect of PA monitoring reactivity,^35^ the participants wore pedometers on the first day right after the initial training. The pedometer-based PA monitoring lasted the whole week, or even longer if the participants wished. To record and evaluate the pedometer-based data and daily PA, the participants had an opportunity to use the Internet-based Indares program that allows evaluation of pedometer data, recorded PA, and other diagnostic tools. These data were not included in the study.

After completion of the research all participants received the following feedback: time data on PA and inactivity, energy expenditure, HR, and step counts. The feedback also included information on load in METs (Metabolic Equivalent of Task) and HR zones, together with clear energy expenditure curves, and a HR curve. Upon completion of the research, individual, and group results were analyzed as a part of biology lessons.

### Procedure

Throughout the day the participants recorded time characteristics before school, during school time according to the timetable of lessons and recess periods, and after school. In the evening, after the devices were removed, the participants recorded the times and types of PA and inactivity/sitting (television, computer, in school, commuting, culture/watching) throughout the whole day. For the purposes of data processing (15-second interval records) we used a specially designed software program, IntPA13, which is available only in the Czech version (http://www.cfkr.eu). The program evaluates data before school, after school, during lessons without PELs, during recess time, during PELs, and in total for school time. The duration of PA and inactivity is indicated in minutes. The rate of PA intensity is determined according to HR from 30% to 100% of in 10% increments, and in METs, in one-MET increments. To identify the HR ranges, we applied a universal formula to calculate maximum HR (for boys, = 220 - age and for girls, = 226 - age). The load zones were classified into low (50-59.9%; <3 METs), moderate (60-84.9%; 3-5.9 METs), and vigorous PA (85-100%; ≥6 METs). The resting metabolic rate was determined according to the following formula: ([473*weight] + [971*height]—[513*age] + 4687])/100,000 for boys and ([331*weight] + [352*height]—[353*age] + 49854])/100,000 for girls. To convert the counts values to kcals/minute values, and then to METs values, the following formula was used: (kcals/minute = .0000191*counts/minute*body mass in kg). Time (in minutes) spent in individual load zones in METs was determined according to individual conversion counts/minute to kcals/minute, ie, according to individual cut points. The sedentary (physical inactivity) cut points were found to be <25 counts per 15 seconds. Physical inactivity is understood as unrecorded change of the “center of gravity” of the body, with up to 100 counts/minute, simply as a “stable body position” in a sitting, lying, or a different body position.

The results of the monitoring included participants who wore the devices for at least 15 minutes prior to school (66 recorded days excluded), for at least 180 minutes during school time without PELs (5 recorded days excluded), for at least 120 minutes after school (8 recorded days excluded), for at least 600 minutes during the whole day (24 recorded days excluded) and for a maximum of 1080 minutes (2 recorded days excluded). One recorded day was obtained from 297 participants, 2 recorded days were obtained from 342 participants, and 3 recorded days were obtained from 47 participants. A total of 1122 monitored school days were used.

### Data Analysis

SPSS 22 and Statistica 12 were used to process the data. For statistical data processing, we used basic statistical characteristics, Kruskal-Wallis test, one-way ANOVA, cross-tabulation tables, Spearman rank correlation, and also effect size coefficients η^2^, ω^2^, and w. The effect size coefficients were considered at the level of .01- < .06 as small effect size, .06- < .14 as medium effect size and ≥.14 as large effect size.

## RESULTS

According to our presumption, both boys and girls participating in PELs reported significantly better results in the indicators of SPA—amount and intensity—compared with nonparticipating individuals. Only in low PA intensity (<3 METs) were the differences not statistically significant (Table2). The differences in physical inactivity (PI) (minutes) and low HR (<50% _-min_) inversely correspond with higher intensity PA characteristics. The highest load of 85-100% of was achieved during SPA by 36.0% (≥8 METs: 72.2%) of individuals participating in PELs, and by only 9.6% (≥8 METs: 19.22%) of nonparticipating individuals.

**Table 2:** School PA in Adolescents Participating and Not Participating in PELs

	Boys				Girls						
	Without PEL (N = 244)		With PEL (N = 95)		Without PEL (N = 547)		With PEL (N = 236)				
Characteristics of PA	Mdn	IQR	Mdn	IQR	Mdn	IQR	Mdn	IQR	H	p	η^2^
kcal/kg/hour (minutes)	0.26	0.23	0.64	0.35	0.22	0.18	0.45	0.23	325.00†‡§∥	.000	.290***
Steps/hour (number)	370.80	287.47	749.23	418.47	369.83	272.75	626.67	353.92	254.20†‡	.000	.227***
Physical inactivity (minute/hour)	42.12	7.88	36.89	8.67	44.73	6.53	39.84	5.35	206.92†‡§	.000	.185***
<3 METs (minute/hour)	15.40	6.88	17.92	7.73	13.50	5.43	17.00	4.90	8.52	.036	.008
3-5.9 METs (minute/hour)	1.53	1.79	3.60	2.22	1.32	1.77	2.64	1.97	31.62†‡	.000	.028*
≥6 METs (minute/hour)	0.09	0.41	0.87	1.35	0.00	0.09	0.33	0.64	78.24†‡§∥	.000	.070**
<50% (minute/hour)	55.13	9.35	44.77	15.93	54.45	8.10	45.27	11.58	232.60†‡	.000	.207***
50-59.9% (minute/hour)	3.99	7.26	10.05	8.66	4.55	5.63	9.28	7.13	173.74†‡	.000	.155***
60-84.9% (minute/hour)	0.44	1.88	5.39	8.24	0.61	2.03	4.92	6.04	254.11†‡	.000	.227***
85-100% (minute/hour)	0.00	0.00	0.00	1.14	0.00	0.00	0.00	0.22	133.18†‡	.000	.119**

PELs, physical education lesson; Mdn, median values; IQR, interquartile ranges; H, Kruskal-Wallis test; η^2^, Cohen's effect size; p, significance level; η^2^,

^*^.01 ≤ η^2^ < .06 small effect size;

^*^^*^.06 ≤ η^2^ < .14 medium effect size;

^*^^*^^*^η^2^ ≥ .14 large effect size.

†Significant difference between groups (1-2).

‡Significant difference between groups (3-4).

§Significant difference between groups (1-3).

||Significant difference between groups (2-4).

Longer recess time (>60 minutes) resulted in a higher volume of SPA in kcal/kg/hour (H = 51.86, p < .001, η^2^ = .066), steps/hour (H = 45.45, p < .001, η^2^ = .058), and in duration of moderate PA (H = 53.14, p < .001, η^2^ = .067), both in girls and boys (Table3). The characteristics of PA intensity according to HR correspond with other PA characteristics; however, the differences are not statistically significant. During recess time, boys spent more time doing intensive PA (≥6 METs) compared with girls.

**Table 3:** School PA According to the Length of Recess Time (School Days Without PELs)

	Boys				Girls						
	Recesses ≤60 minutes (N = 150)		Recesses >60 minutes (N = 94)		Recesses ≤60 minutes (N = 319)		Recesses ≤60 minutes (N = 228)				
Characteristics of PA	Mdn	IQR	Mdn	IQR	Mdn	IQR	Mdn	IQR	H	p	η^2^
kcal/kg/hour	0.23	0.22	0.30	0.28	0.19	0.17	0.27	0.17	51.86†§	.000	.066**
Steps/hour (number)	335.45	277.94	433.45	348.60	336.91	257.54	461.30	266.31	45.45†‡	.000	.058*
Physical inactivity (minute/hour)	42.50	8.11	41.40	7.41	45.28	7.18	43.87	5.18	36.92‡§	.000	.047*
<3 METs (minute/hour)	15.30	7.55	15.71	6.61	13.17	6.05	13.79	4.76	22.78§	.000	.029*
3-5.9 METs (minute/hour)	1.29	1.64	1.82	2.09	1.04	1.41	1.70	1.81	53.14†‡	.003	.067**
≥6 METs (minute/hour)	0.07	0.33	0.14	0.64	0.00	0.09	0.03	0.13	50.54§∥	.000	.064**
<50% (minute/hour)	55.84	7.50	52.46	10.45	54.89	7.82	53.60	9.23	12.45	.006	.016*
50-59.9% (minute/hour)	3.65	5.96	5.45	8.15	4.36	5.09	5.23	6.17	12.37	.006	.016*
60-84.9% (minute/hour)	0.37	1.48	0.75	3.18	0.50	1.99	0.84	1.97	13.97	.003	.018*
85-100% (minute/hour)	0.00	0.00	0.00	0.00	0.00	0.00	0.00	0.00	1.35	.717	.066**

PELs, physical education lesson; Mdn, median values; IQR, interquartile ranges; H, Kruskal-Wallis test; η^2^, Cohen's effect size; p, significance level; η^2^,

^*^.01 ≤ η^2^ < .06 small effect size;

^*^^*^.06 ≤ η^2^ < .14 medium effect size;

^*^^*^^*^η^2^ ≥ .14 large effect size.

†Significant difference between groups (1-2).

‡Significant difference between groups (3-4).

§Significant difference between groups (1-3).

∥Significant difference between groups (2-4).

**Figure 1:**
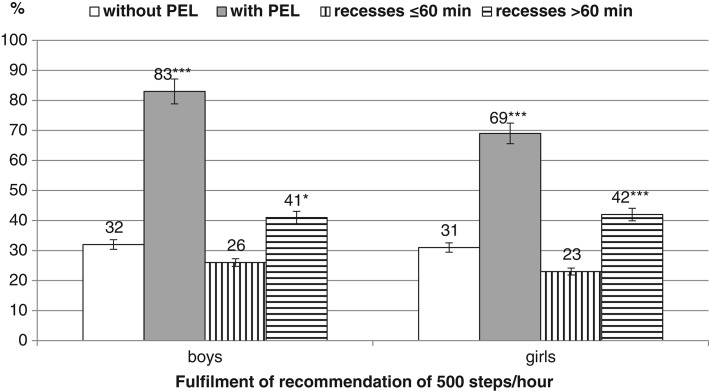
Fulfillment of Recommendation of 500 Steps/Hour During School Time.

The recommendation of 500 steps/hour during SPA (Figure 1) was achieved by 83% of boys participating in PELs compared with 32% of boys not participating in PEL (χ^2^ = 72.06; p < .001; w = .176***). Similarly, the recommendation was achieved by 69% of girls participating in PELs and 31% of girls not participating in PELs (χ^2^ = 101.51; p < .001; w = .339***). With longer recess time the recommendation was achieved by 41% of boys compared with 26% of boys with shorter recess time (χ^2^ = 6.37; p = .012; w = .160***) and by 42% of girls compared with 23% of girls (χ^2^ = 22.04; p = .000; w = .197***).

**Figure 2:**
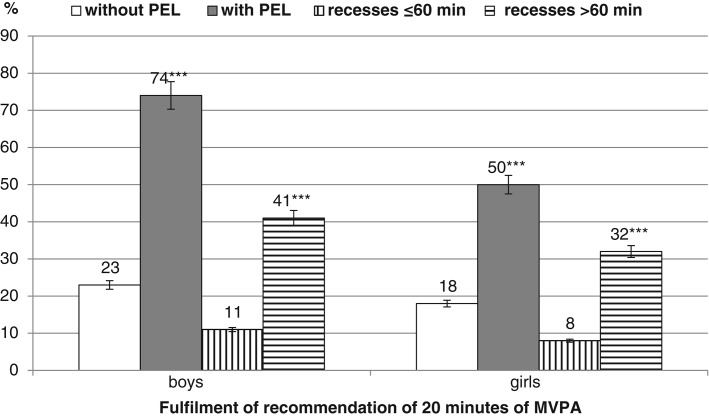
Fulfillment of Recommendation of 20 Minutes of MVPA During School Time.

The recommendation of 20 minutes of MVPA during SPA (Figure 2) was achieved by 74% of boys participating in PELs compared with 23% of boys not participating in PEL (χ^2^ = 74.36; p < .001; w = .183***). Similarly, the recommendation was achieved by 50% of girls participating in PELs but only 31% of girls not participating in PELs (χ^2^ = 101.51; p < .001; w = .116**). With longer recess time the recommendation was achieved by 41% of boys compared with 11% of boys with shorter recess time (χ^2^ = 29.72; p < .001; w = .109**) and by 32% of girls compared with 8% of girls with shorter recess time (χ^2^ = 54.39; p < .001; w = .091**).

Boys with over 500 steps/hour during school time achieved an average of 12,171 steps/day for the whole day (other boys 8820 steps/day) and girls 12,035 steps/day (other girls 9592 steps/day) (F = 52.21; p < .001; ω^2^ = .120). The daily recommendation of 11,000 steps/day was achieved by 54.14% of boys who had more than 500 steps/hour during school time (25.27% of other boys) (χ^2^ = 29.62; p < .001; w = .296***) and 56.93% of girls (31.49% of other girls) (χ^2^ = 50.72; p < .001; w = .255***). Boys with over 20 minutes of MVPA during school time achieved an average of 34.91 minutes of MVPA/day for the whole day (other boys 25.74 minutes) and girls 32.10 minutes (other girls 23.09 minutes) (F = 47.63; p < .001; ω^2^ = .150). The daily recommendation of 60 minutes of MVPA/day was achieved by 62.70% of boys who had more than 20 minutes of MVPA during school time (37.56% of other boys) (χ^2^ = 20.09; p < .001; w = .243***) and 59.91% of girls (29.33% of other girls) (χ^2^ = 62.38; p < .001; w = .282***).

The average proportion of school PA in overall daily PA was 31.8% in boys (30.6% in girls) according to step counts, 29.9% in boys (26.7% in girls) according to MVPA minutes, 31.1% in boys (2.6% in girls) according to minutes of PA of intensity > 60, and 37.8% in boys (33.9% in girls) according to minutes of overall PA.

## DISCUSSION

As expected, the most significant role in school PA is played by PELs, a finding confirming results of previous studies.^18^,^36^ In our research it was confirmed that PELs have an identically significant effect for boys and for girls, both in terms of PA volume and intensity. It is also important that during PELs, 90.5% of boys and 78.8% of girls achieved the high-intensity zone load (≥85% or ≥6 METs), which is also important in terms of compensating for the stress of school and the educational process on mind and body. The recommendation^4^,^37^ that 50% of time during PELs be made of MVPA was achieved by 46.3% of boys and 35.2% of girls. A number of other studies imply that during regular PE classes students spend between 27% and 47% of class time engaged in MVPA.^13^ Surprisingly, the fulfillment of this recommendation for PELs was observed in less than 5% of children.^38^ These differences confirm the difficulty of defining recommendations that respects or addresses various aims, different contents, and other variables. If time for PELs includes 50% of PA of nonspecified intensity, 87.4% of boys and 81.8% of girls would fulfill this lower indicator in our research. Regarding the various concepts of school PE, including objectives and content of lessons, a minimum of 50% of PA in adolescents in any type of PEL is considered sufficient and also corresponds with the recommendation first raised in *Healthy People 2010*.^39^

Most studies focus on the proportion of PELs and PA in all-day PA. The proportion of physical education class PA in daily PA was 8.7-23.7% in boys and 11.4-17.2% in girls.^33^ In 9- to 11-year-old children, PELs represented 16.7% of overall daily step counts, 25.1% of overall MVPA duration, and 24.1% of MVPA heart-rate response.^40^ In our research the proportion of PELs in the daily step counts in boys was 14.33% and 11.98% in girls. With respect to MVPA, duration was 16.54% in boys and 13.38% in girls. However, the role of PE is may be more far reaching that just contributing to overall PA in adolescents. Short- and long-term health and fitness effects of physical education should be a high priority for future research.^15^

Longer aggregate recess time had a positive effect on SPA, particularly in terms of PA volume. However, it was confirmed that not even longer aggregate recess time could substitute for PELs in SPA in terms of intensive PA. In the MVPA zone girls spent 8.0% and boys 9.7% of recess time, which is significantly less compared with other findings concerning children. During recess time 38% of 8- to 10-year-old girls and 31% of boys were engaged in MVPA;^41^ in a different study, it was 20% of 8- to 11-year-old girls and 28% of boys.^38^ The recommendation of 50% of MVPA of the total recess time for children^42^ was achieved by only 1.9% of boys and 1.4% of girls in our sample. For secondary schools adolescents a more appropriate recommendation is 50% of any PA of the total recess time, ie, PI/PA ratio of 1:1. In our research, 57.5% of boys and 55.4% of girls would achieve such a recommendation.

As expected, boys spent more time engaged in vigorous PA during recess time compared with girls, which corresponds with other results.^25^,^26^,^38^ Recess time covered 8-11% and lunchtime 15-16% of total steps per day^33^ while in our research the combination of those 2 sessions was 13.42% in boys and 14.37% in girls. In terms of MVPA, recess time covered 17.9% of all-day MVPA in boys and 15.5% in girls;^23^ in one longitudinal study, after 5 years it was 10.7% in 10- to 12-year-old boys and 6.6% in girls.^26^ In our research the average proportion of recess time in all-day MVPA was 8.8% in boys and 8.1% in girls for those with shorter aggregate recess time; in boys with longer aggregate recess time, it was 19.0% and 17.0% in girls.

The proportion of SPA in overall daily PA was 31.4% according to step counts, 29.0% according to MVPA minutes, 28.8% according to minutes of PA of intensity > 60, and 36.1% according to minutes of overall PA. These results correspond with the findings^10^ that school MVPA in secondary schools represents 23% of overall weekly MVPA. In one study assessing overall daily step counts, the proportion of SPA was 42-49% in boys and 41-47% in girls.^33^ In our research, the average proportion was 31.8% in boys and 30.6% in girls. Searching for new ways of increasing the proportion of SPA in daily and weekly PA is still timely and relevant. Intervention-based evidence is especially beneficial. Interventions under “active school day” increased student MVPA by 24% and vigorous PA by 46% and decreased sedentary time by 6%.^43^ Further intervention-based evidence is required to explain how SPA contributes to more daily PA, particularly in secondary schools adolescents.

Only 35 European studies out of 131 declare achievement of daily PA recommendations for young people.^29^ Therefore, an effort to define recommendations for SPA in adolescents is legitimate and can contribute to more PA overall in school children. The recommendation of 500 steps/hour (3000 steps/school time) based on our research should be used by school management and teachers to develop school environments and organization that would require students to engage also in short-term work-related PA, transfers between places, changes in body position, both during lessons, and during recess time. Low PA and short episodes of MVPA have health benefits and should be accounted for in PA and health research.^44^ The recommendation of 3000 steps/school time is also supported by unpublished results of our other research studies focusing on SPA monitoring in adolescents by means of pedometers. During school time, boys (N = 549) performed on average of 3738 steps (SD = 1911; median [Mdn] = 3536; interquatile range [IQR] = 2517); girls (N = 1117) performed on average 3231 (SD = 1534; Mdn = 2574; IQR = 1713).

The fulfillment of the recommendation of 20 minutes of MVPA in school is a significant foundation for achieving the daily PA recommendation. Approximately 62.70% of boys and 59.91% of girls in our research who had more than 20 minutes of MVPA in school achieved the daily recommendation of 60 minutes of MVPA/day (versus other boys with 37.56% and girls with 29.33%). A research study performed in 5 European countries revealed that only 16.8% of boys and 4.6% of girls achieved the recommendation of 60 minutes of MVPA/day; however, the measured age group consisted of 11- to 12-year-olds.^45^ In younger children we would expect a higher proportion of fulfillment of the PA recommendation compared with older individuals. These comparisons highlight the limitations of current PA monitoring by means of accelerometers in terms of the level of PA or achievement of PA recommendations.

The selection of the load intensity zones according to HR in 10% MHR proved to be sufficient. The load zones in METs (for SPA) were determined according to the most commonly used classification^46^ at the lowest level. In the context of SPA, physical load in the zone of MVPA ≥60% significantly correlates with physical load ≥3 METs (r_s_ = .394) and similarly in the zone of moderate PA (60-84.9%; 3–5.9 METs) (r_s_ = .431). At the same time it needs to be respected that PA monitoring by means of accelerometers at a population level is a better predictor for low loads of PA than HR record.^47^

PA and HR monitoring using the ActiTrainer device allowed objective measurement of physical load in natural school conditions. In terms of objectivity, a combination of PA and HR monitoring and PA record appears to be the most appropriate method of PA monitoring in natural school conditions. Furthermore it is obvious that registering the participants in the Internet-based Indares program contributed to better documentation of personal data and promotion of positive changes in school lifestyle. Overall, 79.9% of boys and 64.4% of girls used the links between PA monitoring and the Internet-based Indares program.

Our research experience with other schools shows that the recess times in the educational system are well established; nevertheless it is necessary to persuade educational/school authorities to abbreviate the last lessons of the school day and to stop reducing the duration or frequency of recess time. Recess should offset the mental or intellectual demands of cognitively tailored lessons and create an emotional state that is conducive to subsequent learning of the academic subjects. Occasional PA in the classroom to relieve the tedium or stress of the school day, as well as reduction in the amount of sedentary behavior during recess times should be emphasized. Secondary schools also should strive to meet the PA standards of the school system by implementing periods of exercise in lessons other than PE.

A particular strength of this study includes its HR and PA monitoring using a single device—ActiTrainer, which makes an objective assessment of PA intensity and comparison of load expressed in HR, minutes of PA, step counts and METs. Another strength lies in the high number of days of all-day HR and PA monitoring in adolescents in natural school settings without interference with the school program. To the best of our knowledge, no similar studies have been reported to date in the school health literature.

### Limitations

The most significant limitation of the study is the difficult all-day HR monitoring, particularly wearing chest belts with a HR sensor. With respect to possible complications with the HR sensor the participants avoided swimming on the monitoring days. The quality of the study would improve by objective determination of as opposed to using a universal calculation formula. A certain limitation is the absence of objectively determined physical performance of the participants, which influences the speed of changes in HR and absence of objectively assessed positive or negative stress influences on HR during lessons. These PA monitoring aspects need to be a focus in future research studies.

### Conclusion

PA lessons have stronger effect on a physically active lifestyle in boys and girls as opposed to longer aggregate recess time. Longer aggregate recess time contributed to an increased volume of SPA in both sexes; however, this cannot be a substitute for PELs in school. School PA has an irreplaceable position in overall daily PA in adolescents.

## IMPLICATIONS FOR SCHOOL HEALTH

The following guidelines for a physically active lifestyle during school time are ones that secondary schools should promote among adolescents:

- Achievement of at least 500 steps/hour (ie, approximately 3000 steps/school time);
- Achievement of at least 20 minutes MVPA/school time;
- Achievement of at least one considerable episode of high PA intensity—if PELs or a different physically oriented organizational form is involved in the school program;
- At least 50% of recess time should be filled with PA; and
- SPA should cover at least 25% of overall school time.

These guidelines should present a challenge leading to changes in the traditional organization of lessons and promotion of healthy and active school lifestyles among students and teachers.

Our study points out the importance of adopting an active school lifestyle in adolescents as a basis for future employment. In school lifestyle and on school days, PELs are difficult to replace, especially in terms of intensive PA, which emphasizes the significance of an extracurricular program and other organized forms of PA after school. For school health, however, equally important is any health-oriented PA throughout the educational process using appropriate teaching styles. Immediate individual and group feedback on the level of PA together with PA records and analysis using an Internet-based program allow teachers to perform evidence-based cross-curricular integration and health education of adolescents.

### Human Subjects Approval Statement

This study was approved by the Institutional Review Board of the Faculty of Physical Culture, Palacký University Olomouc under reference number 24/2012, and parental consent to participate in the study was obtained for all adolescents in the sample.
